# Philadelphia Times and Register

**Published:** 1889-06

**Authors:** 


THE CONSOLIDATION of the *Philadelphia Medical Times*, the *Medical Register*, and the *Dietetic Gazette*, is to be commended, on the ground that the country is overflowing with medical magazines, and any such move that tends to reduce the number and improve the quality is in the direction of gain to the medical journal readers. *The Times and Register*, which will henceforth be the designation of the consolidated journal, will be issued as a weekly under the editorial management of Dr. William F. Waugh,—an indication of a successful career for the new enterprise, which we sincerely hope may be vouchsafed to it.

